# Identification of self- and pathogen-targeted miRNAs from resistant and susceptible *Theobroma cacao* variety to black pod disease

**DOI:** 10.1038/s41598-024-53685-x

**Published:** 2024-02-08

**Authors:** Popi Septiani, Yonadita Pramesti, Devi Ulfa Ningsih, Sulistyani Pancaningtyas, Karlia Meitha

**Affiliations:** 1 School of Life Sciences and Technology, Institut Teknologi Bandung, Jl. Ganesha No. 10, Bandung, 40132 Indonesia; 2 Indonesian Coffee and Cocoa Research Institute (ICCRI), Jl. PB. Sudirman 90, Jember, 68118 Indonesia

**Keywords:** Biotic, Plant molecular biology, Biotechnology, Molecular biology, Plant sciences

## Abstract

Cacao (*Theobroma cacao*) is a highly valuable crop with growing demand in the global market. However, cacao farmers often face challenges posed by black pod disease caused by *Phytophthora* spp., with *P. palmivora* being the most dominant*.* Regulations of various gene expressions influence plant resistance to pathogens. One mechanism involves targeting the mRNA of virulence genes in the invading pathogens, suppressing their infection. However, resistance also could be suppressed by plant-derived miRNAs that target their own defence genes. The objective of this study is to identify differentially expressed miRNAs in black pod-resistant and susceptible cacao varieties and to predict their targets in *T. cacao* and *P. palmivora* transcripts. Extracted miRNA from resistant and susceptible varieties of *T. Cacao* was sequenced, identified, and matched to host and pathogen mRNA. In total, 54 known miRNAs from 40 miRNA families and 67 novel miRNAs were identified. Seventeen miRNAs were differentially expressed in susceptible variety compared to resistant one, with 9 miRNAs upregulated and 8 miRNAs downregulated. In *T. cacao* transcripts, the upregulated miRNAs were predicted to target several genes, including defence genes. The suppression of these defense genes can lead to a reduction in plant resistance against pathogen infection. In *P. palmivora* transcripts, the upregulated miRNAs were predicted to target several genes, including *P. palmivora* effector genes. In the future, limiting expression of miRNAs that target *T. cacao*’s defence genes and applying miRNAs that target *P. palmivora* effector genes hold promise for enhancing cacao plant resistance against *P. palmivora* infection.

## Introduction

The decrease in cacao (*Theobroma cacao*) productivity in Indonesia has significantly affected plantations and associated industries. In 2015, Indonesia ranked third globally as a cacao producer, following Côte d'Ivoire and Ghana. However, over the past seven years, Indonesia's cacao production has experienced a decline, leading to a seventh position among the world's cacao producers. Nonetheless, Indonesia remains the largest cacao producer in Asia, contributing to a total of 5.7% of the Asian production^1^.

Ariningsih et al.^2^ attributed the decline in cacao productivity in Indonesia to factors such as inadequate plant maintenance, pests and diseases. Among the diseases affecting cacao, the most prominent is black pod disease (BPD), caused by oomycete infections from the Phytophthora genus^3^. Four species of Phytophthora have been identified as causative agents of BPD. These include *Phytophthora palmivora*, *Phytophthora megakarya*, *Phytophthora capsici*, and *Phytophthora citrophthora*. Among these species, *Phytophthora palmivora* is recognized as the most destructive, causing a global production reduction of 44%^4^. In some regions of Indonesia, losses caused by this pathogen have reached 30–50%^5^.

The most common approach for BPD control in cacao is the periodic application of chemical fungicides. However, these chemicals pose environmental challenges like soil and water pollution. Additionally, fungicides can negatively impact human health and beneficial soil microorganisms^6^. Therefore, alternative strategies with limited detrimental environmental effects, such as biocontrol methods, are required. Another approach involves utilizing cacao varieties that exhibit resistance to BPD. However, resistant varieties do not always possess other desirable traits, such as high fruit yield and shorter life cycles. Consequently, breeding techniques are necessary to introduce resistance to *P. palmivora* in susceptible varieties that already display superior phenotypic characteristics.

Plant susceptibility is known to be influenced by gene expression regulation. One mechanism involves the production of micro-RNAs (miRNAs) that specifically target genes associated with the defense system. MiRNAs are short non-coding sequences of RNA that once leaving the nucleus could bind to their complementing mRNAs. Thereby initiating RNA interference (RNAi) and suppressing the expression of their defense system genes^7^. According to Yang^8^, a significant number of miRNAs target resistance genes, particularly NBS-LRR genes, which play a crucial role in pathogen recognition in plants due to their nucleotide-binding site (NBS) and leucine-rich repeat (LRR) domains. For instance, the heightened susceptibility of potato plants to *Verticillium dahliae* infection resulting from the overexpression of stu-miR482e can be attributed to the increased suppressive impact induced by stu-miR482e on the NBS-LRR genes responsible for disease resistance^9^. These findings highlight the potential of eliminating these miRNAs to enhance plant resistance to infection.

In addition, cross kingdom RNAi is proposed as one of plant defense mechanisms against pathogen infection, which is carried out by transferring small regulatory RNAs into pathogens that attack the plant. This process leads to the silencing of virulence genes in the pathogen, thereby suppressing pathogen infection^10^. miRNAs exhibit environmentally friendly characteristics and have high specificity to their target. Koch et al.^11^ demonstrate that applying CYP3-dsRNA that targets *Fusarium graminearum* CYP3 gene through spraying or spray-induced gene silencing (SIGS) can effectively repress the infection of *F. graminearum* on barley. These findings highlight the potential of utilizing miRNAs as a biocontrol strategy against pathogen infection.

This study aims to identify differentially expressed miRNAs in black pod disease-resistant and susceptible cacao varieties. Additionally, the study aims to identify the targets of these miRNAs within the *T. cacao* defense system gene and *P. palmivora* virulence gene. In future applications, the elimination of miRNAs that target *T. cacao* defense system genes is expected to restore the expression levels of these genes, enhancing resistance against *P. palmivora* infection and also utilizing miRNAs that target the virulence genes of *P. palmivora* as a biocontrol is also expected to suppress its infection effectively.

## Results

### Resistance and Susceptible varieties of *T. cacao* and Disease Scoring

**Figure 1:**
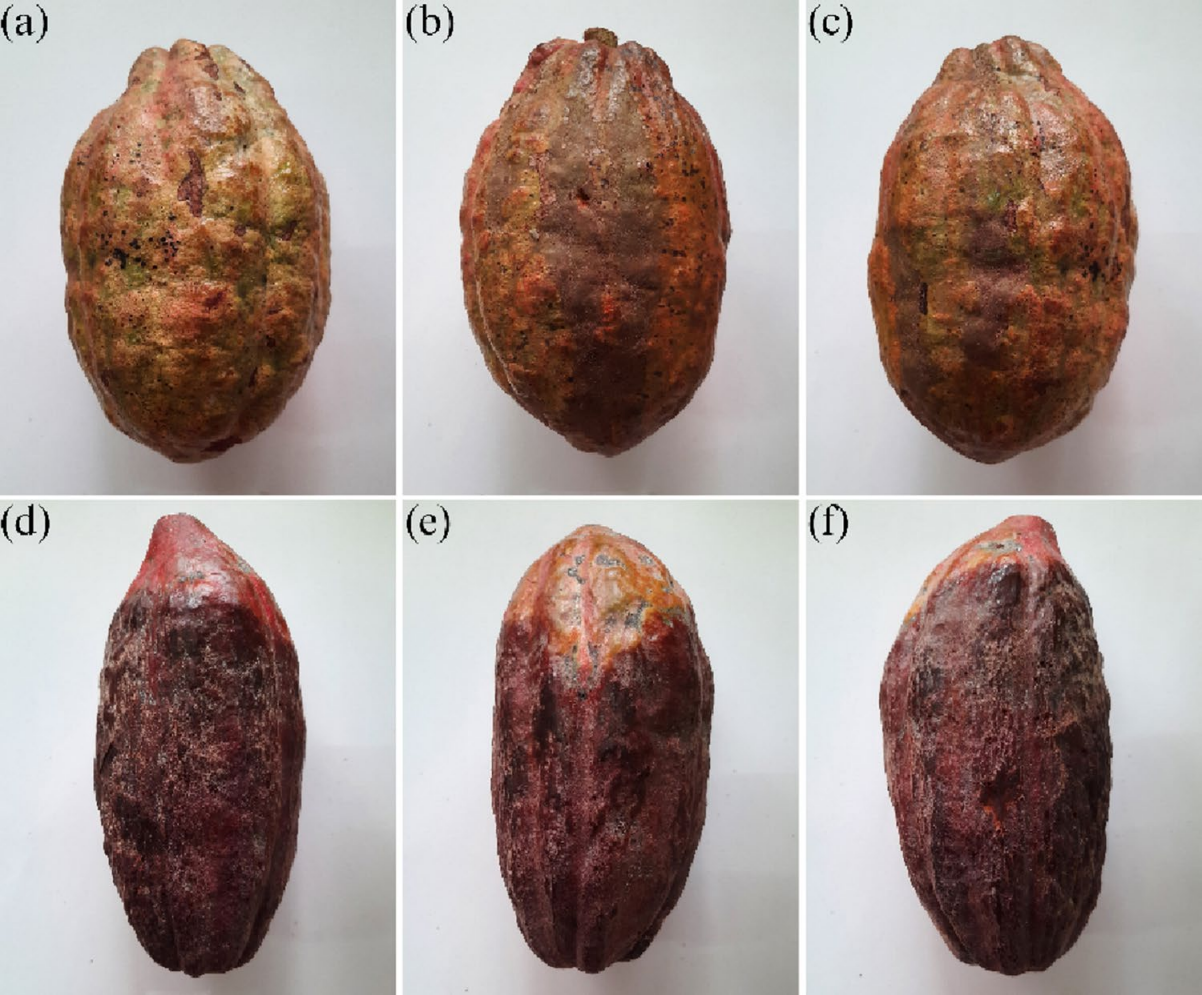
*T. Cacao* pod used in miRNA extraction, the images of each pod were captured from three different angles. (**a**–**c**) Resistant (TSH858) and (**d**–**f**) susceptible (Sulawesi 1) varieties.

In this research, *T. cacao* pod from resistant (healthy) TSH858 and susceptible (infected) Sulawesi 1 varieties (Fig. 1) were respectively collected from 9 years old tree at the cacao plantation of the Indonesian Coffee and Cocoa Research Institute with geographical coordinate of 8°15′31.7′′ S 113°35′50.1′′ E in Rambipuji sub-district of Jember, East Java Indonesia. The sampling was conducted during the dry season in July 2022, characterized by an average temperature of 26.5 °C, humidity of 90.52%, solar radiation of 150.94 W m^−2^, light intensity of 64.11%, wind speed of 0.23 m s^−1^, zero precipitation, and an evapotranspiration rate of 2.94 mm/day. Disease scoring was performed to assess the extent of infection. Resistant cacao pod exhibited no signs of rot, whereas the susceptible cacao pod displayed one rotten zone with an area of 720.63 cm^2^ (Table 1).

**Table 1:** Disease scoring of samples used in miRNA extraction.

Sample	Pod length (cm)	Pod circumference (cm)	Zone	Diameter 1 (cm)	Diameter 2 (cm)	Area (cm^2^)
Resistant	17.7	29.8	–	–	–	–
Susceptible	19.2	26.7	1	30.8	29.8	720.63

To confirm the presence of *P. palmivora*, PCR amplification was conducted using specific primers targeting the ITS2 and 5.8S rDNA regions of the *P. palmivora* genome. Subsequent sequencing of the amplified products indicated the presence of *P. palmivora* in the susceptible cacao pods, as shown by the aligned sequences to the ITS2 and 5.8S rDNA genes of *P. palmivora* in NCBI database, while the resistant cacao pods did not exhibit any traces of *P. palmivora* (Supplementary information, Table 1).

### Sequencing of small RNA

miRNA extraction was performed on the samples by using a microRNA extraction kit. The extracted miRNAs were then subjected to sequencing analysis to elucidate their expression profiles. The results are displayed in (Supplementary information, Table 2). Raw reads from the sequencing results showed good quality, as indicated by the absence of low-quality reads across all four samples. The presence of reads with an N exceeding 10% was only observed in sample S2, with a minimal percentage of 0.01%. Low-quality reads were removed to ensure data quality, resulting in a range of clean reads from 2,974,499 to 11,124,113. Subsequently, a length-based filter was applied, selecting reads with lengths between 18 and 28 base pairs. These filtered results provided the final set of clean reads for further analysis. Final clean reads obtained for samples R1, R2, S1, and S2 are 10,685,251; 2,856,473; 3,861,549; and 4,134,082, respectively.

**Table 2:** The 10 most abundant known & novel miRNAs identified from *T*. *Cacao* libraries.

miRNA ID	Sequence (5′–3′)	Chromosome	Start position	End position	Strand	R1	R2	S1	S2
Tca-MIR159d	TTGGACTGAAGGGAGCTCCT	NC_023616.1	25272801	25272821	+	48,938	22,272	357	528
Tca-MIR3954a	TTGGACAGAGTAATCACGGTCG	NC_023622.1	30293905	30293926	−	2992	182	111	311
Tca-MIR482a	TCTTCCCAACACCTCCCATACC	NC_023624.1	22810014	22810035	+	20,440	950	8	20
Tca-MIRN9	TTCACTTCTCTGATTTGGGCGA	NC_023619.1	19178360	19178381	−	9948	341	2	19
Tca-MIR472a	TCTTCCCTACTCCACCCATCCC	NC_023621.1	40294561	40294582	−	6525	490	3	21
Tca-MIRN7068a	ACTCTCTTTCGAAGGCTTCAAG	NC_023625.1	35431242	35431263	−	5592	445	2	24
tcc-miR162	TCGATAAACCTCTGCATCCAG	NC_023623.1	22612635	22612655	−	1260	754	23	18
tcc-miR166a	TCGGACCAGGCTTCATTCCCC	NC_023623.1	28905616	28905636	−	2302	183	20	30
Tca-MIR159b	TTGGACTGAAGGGAGCTCCC	NC_023623.1	32655000	32655019	−	687	111	21	61
tcc-miR403a	TTAGATTCACGCACAAACTCG	NC_023622.1	32849355	32849375	−	3260	212	3	16
Novel-miR59	CAGTTTAATATCTGATACATG	NC_023625.1	4587917	4587943	−	659	26	1183	1418
Novel-miR1	TTGCCGAGTCCGCCCATGCCTA	NC_023624.1	22796221	22796242	+	31,310	2312	84	128
Novel-miR6	GATAAATCATTGCAATTGTTG	NC_023619.1	21211154	21211177	+	520	202	305	322
Novel-miR42	GATAACATCATAGGATTA	NC_023622.1	29085332	29085349	+	788	100	49	237
Novel-miR18	GCGGCGATGATGATTGAAAA	NC_023618.1	883902	883920	+	55	10	34	50
Novel-miR5	CTTTTAACCGACTTTTGCACTCCC	NC_023617.1	4348336	4348356	−	1914	285	9	10
Novel-miR2	TTGCCGGCTCCGCCCATTCCAA	NC_023624.1	22809818	22809839	+	3627	117	0	3
Novel-miR4	ACAGGGAACAGGCTGAGCATG	NC_023625.1	34189717	34189737	−	1180	107	13	8
Novel-miR34	AATGTAAATCTGAACAATTC	NC_023619.1	572382	572402	+	27	8	5	20
Novel-miR39	AGCTCCTGAGATATGCAAGC	NC_023623.1	4147363	4147383	−	816	70	0	0

The clean reads obtained were subsequently aligned to the cacao genome to determine their genomic positions. The mapping results revealed that in samples R1, R2, S1, and S2, a total of 3,999,744; 1,619,130; 1,218,792; and 1,192,304 reads, respectively, were successfully mapped to the cacao genome. miRNA identification revealed a range of 647–112,728 reads for known miRNAs and 1703–46,193 reads for putative novel miRNAs.

### Identification of known and novel miRNA

Known miRNA identification was conducted by aligning the reads against the miRNA cacao database derived from miRBase and PmiREN. In *T. cacao* susceptible varieties, a total of 51 known miRNAs were successfully identified, whereas in *T. cacao* resistant varieties, 33 known miRNAs were identified. The reads that were unassigned to known miRNAs were further subjected to novel miRNA prediction using MirDeep2. The prediction analysis revealed the presence of 67 novel miRNAs in *T. cacao* resistant variety and 28 novel miRNAs in *T. cacao* susceptible one (Table 2 and Supplementary information, Table 3).

**Table 3:** List of differentially expressed miRNA and their fold change value, miRNAs with log2FoldChange > 1 were up-regulated and miRNAs with log2FoldChange < 1 were down-regulated.

miRNA ID	log2FoldChange	padj	Regulation
Novel-miR59	7.6892	5.85E−15	Up
Tca-MIR3627a	6.04285	7.98E−05	Up
Novel-miR18	5.52714	1.96E−08	Up
Novel-miR6	4.48371	6.76E−06	Up
Novel-miR34	4.39792	0.00029	Up
Novel-miR42	3.66243	0.00077	Up
tcc-miR167a	3.49287	0.00899	Up
tcc-miR156b	3.17673	0.00426	Up
Tca-MIR3954a	2.70891	0.01165	Up
Tca-MIR472a	− 2.6597	0.02425	Down
tcc-miR396c	− 2.8984	0.01232	Down
Tca-MIRN9	− 3.1733	0.01232	Down
Tca-MIR482a	− 3.8652	0.00059	Down
Novel-miR2	− 4.4923	0.0039	Down
Novel-miR9	− 5.0869	0.01232	Down
Novel-miR39	− 5.7109	0.00426	Down
tcc-miR399a	− 6.1748	0.00215	Down

**Figure 2:**
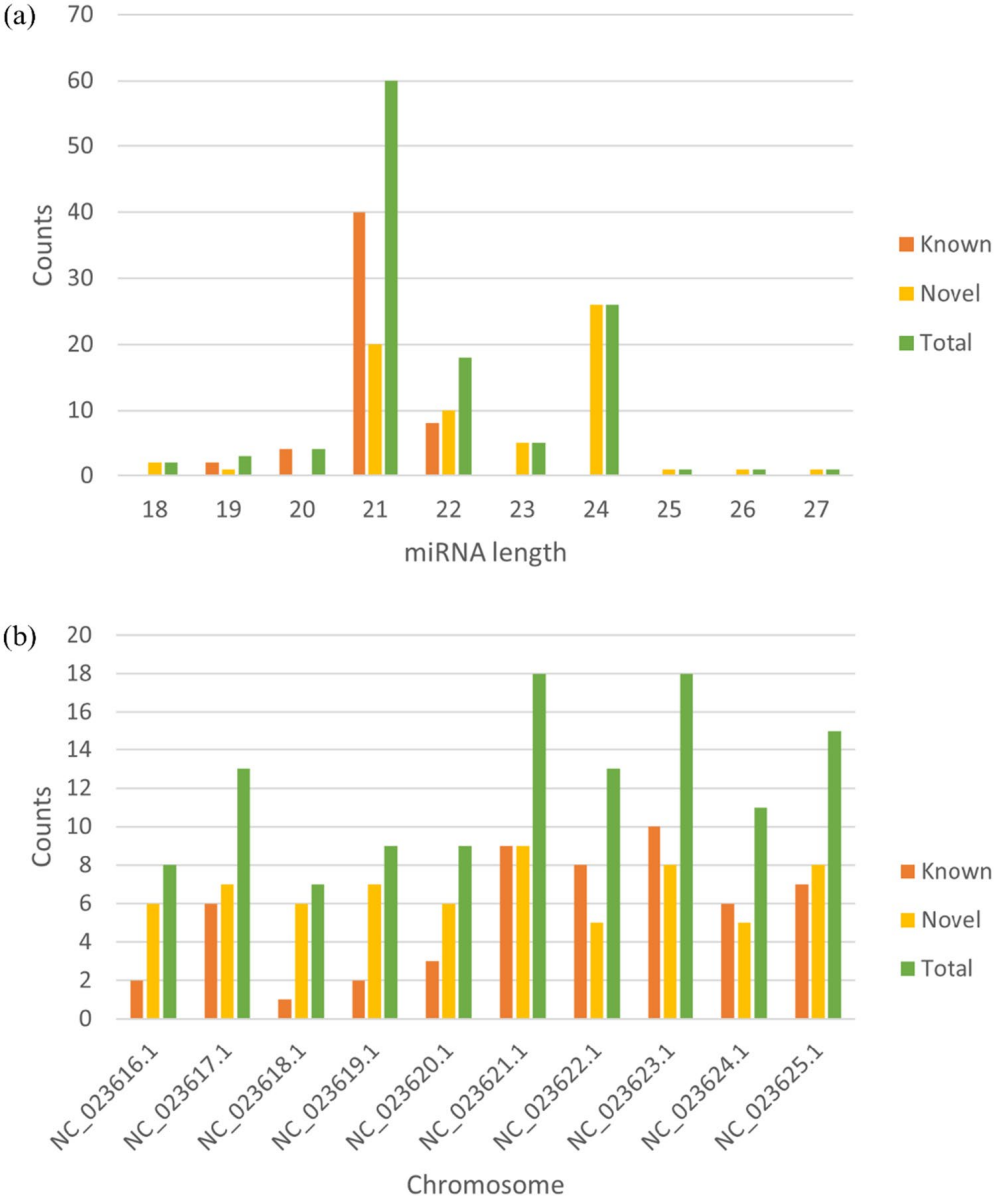
(**a**) Distribution of known and novel miRNAs length; and (**b**) Distribution of miRNAs in *T. cacao* chromosomes.

We found that the length of the identified miRNAs ranged from 18 to 27 nucleotides, with the majority (60 miRNAs) having a length of 21 nucleotides. Following this, miRNAs with a length of 24 nucleotides ranked second with the highest count of 26 miRNAs, while miRNAs with a length of 22 nucleotides accounted for 18 miRNAs. miRNAs with lengths beyond the categories were present in fewer numbers, with less than 5 miRNAs each (Fig. 2a). The identified known and novel miRNAs were also distributed across 10 chromosomes within the cacao genome. Chromosomes NC_023621.1, NC_023633.1, and NC_023625.1 contained the highest abundance of miRNAs, exceeding 15 in number, while the remaining 7 chromosomes, NC_023616.1, NC_023617.1, NC_023618.1, NC_023619.1, NC_023620.1, NC_023622.1, and NC_023624.1, contained fewer than 15 miRNAs (Fig. 2b).

### Analysis of differentially expressed miRNA

**Figure 3:**
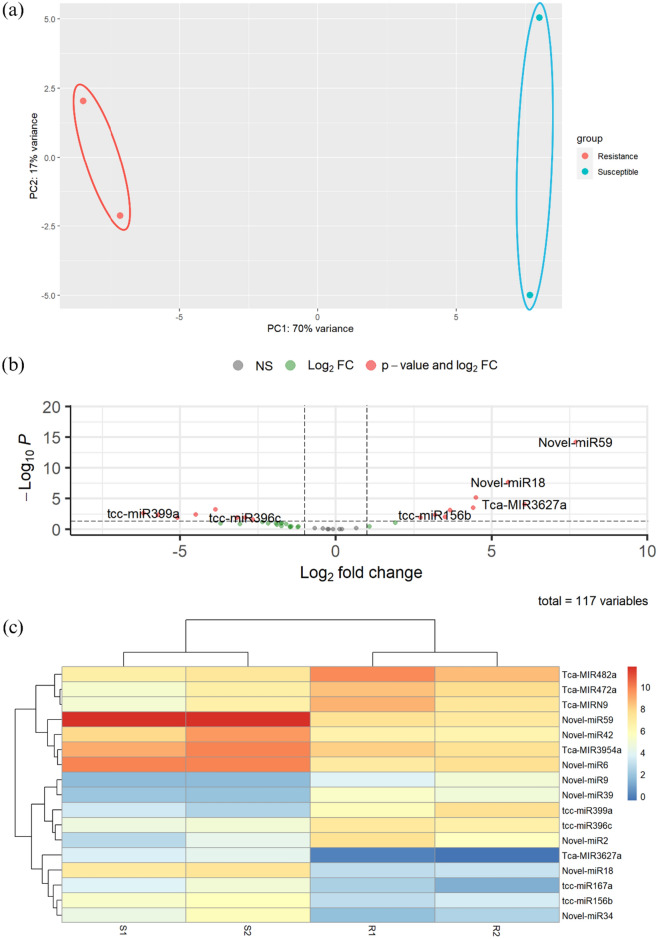
Visualization of differentially expressed miRNAs (**a**) PCA plot visualization of the two samples of resistant and susceptible varieties shows the clustering differences between the two groups of samples, (**b**) Volcano plot shows differentially expressed miRNAs are represented by red plots, and (**c**) Heatmap illustrating differentially expressed miRNAs with a gradient of red color indicating miRNA upregulation and a gradient of blue color indicating miRNA downregulation.

The PCA plot visualization (Fig. 3a) shows the grouping of miRNAs into distinctive clusters. miRNAs from resistant variety of TSH858, R1 and R2, clustered together in the red-colored plot, while miRNAs from susceptible variety of Sulawesi 1, S1 and S2, clustered in the blue-colored plot. This indicates a distinct separation between the resistant and susceptible samples based on their miRNA quantification. The PCA plot provides insights into the differences in principal component values within the two sample groups under investigation.

As much as 17 miRNAs with *p*adj value < 0.05 (Fig. 3b) were identified as differentially expressed out of a total of 117 miRNA normalization variables. The visualization of the volcano plot indicates these miRNAs with red plots. Out of them, nine miRNAs were upregulated while the other eight were downregulated. The most upregulated miRNA was Novel-miR59 with a log_2_FoldChange value of 7, indicating a significant increase in expression. On the other hand, tcc-miR399a showed the most pronounced decrease in expression with a log_2_FoldChange value of − 6, as shown in Table 3 and Supplementary information, Table 4. As depicted in Fig. 3c, the expression pattern of miRNAs exhibited a high degree of consistency in both the resistant and susceptible replicates.

**Table 4:** Differentially expressed miRNAs that target both their own transcripts and genes in *P. palmivora* without contradicting each other.

miRNA ID		Regulation	Target on *Theobroma cacao* transcripts		Target on *Phytopthora palmivora* transcripts	
			Target prediction	Protein function	Target prediction	Protein function
Tca-MIR3954a		Up	Protein chromatin remodeling 19 (LOC18609933) mRNA	Plant pathogen resistance	CAMK/CDPK protein kinase complete cds	Protein phosphorilation
Novel-miR34		Up	Cellulose synthase-like protein D3 (LOC18599512) mRNA	Synthesis of a cell wall polysaccharide essential	CMGC/DYRK protein kinase complete cds	Protein phosphorilation
tcc-miR396c		Down	G-type lectin S-receptor-like serine/threonine-protein kinase LECRK1 (LOC18611782) mRNA	Involved in innate immunity	Pre-mRNA-splicing factor SYF1-like protein (PITG_11158) mRNA, complete cds	Pre-mRNA splicing
Tca-MIR472a		Down	Putative disease resistance protein At3g14460 (LOC108662475) mRNA	Potential disease resistance protein	Transmembrane protein, putative (PITG_19640) complete cds	Transmembrane protein
Tca-MIR482a		Down	Probable disease resistance protein At1g12280 (LOC18595262) mRNA	Potential disease resistance protein	Cytochrome oxidase subunit I (coxi) gene, partial cds; mitochondrial	Cellular respiration
Novel-miR6		Up	RING finger protein 5 (LOC18591332) mRNA	Protein ubiquitination	Secreted RxLR effector peptide protein, putative (PITG_13593) complete cds	Effector that acts as an elicitor of hypersensitive response (HR)
tcc-miR399a		Down	Probable ubiquitin-conjugating enzyme E2 24 (LOC18596849) mRNA	Protein ubiquitination	Secreted RxLR effector peptide protein, putative (PITG_14984) complete cds	Effector that acts as an elicitor of hypersensitive response (HR)

Eight of the 17 differentially expressed miRNAs are putative novel miRNAs: Novel-miR2, Novel-miR6, Novel-miR9, Novel-miR18, Novel-miR24, Novel-miR39, Novel-miR42, and Novel-miR59. The remaining miRNAs are known and can be retrieved from both the miRBase and PmiREN databases.

### Target prediction on *T. cacao* and *P. palmivora* genome

Known miRNAs and novel miRNAs were subjected to target gene prediction in the transcripts of *T. cacao* and *P. palmivora*. psRNATarget analysis revealed that, in the susceptible variety, 54 known miRNAs and 67 novel miRNAs were predicted to regulate up to 14,716 genes in the susceptible *T. cacao* genotype. Among these, 12,398 genes were regulated through cleavage and 2318 genes were regulated through translational repression. In the 9 upregulated miRNAs, several miRNAs exhibit a broad range of target genes. For instance, tcc-miR156b displaying the highest number of target genes with 188 target genes. The following tcc-miR156b are Novel-miR34, tcc-miR167a, Novel-miR18, Novel-miR6, Novel-miR59, Tca-MIR3954a, Tca-MIR3627a, and Novel-miR42 that target 158, 157, 156, 154, 147, 135, 116, and 4 genes, respectively.

Among the targets of upregulated miRNAs in *T. cacao* transcripts, 17 disease-resistance genes are regulated by tcc-miR156b, tcc-miR167a, Novel-miR6, Novel-miR34, and Novel-miR59. Furthermore, Tca-MIR3627a, Novel-miR6, Tca-MIR3954a, and Novel-miR34 were predicted to target 7 L-type lectin-domain containing receptor kinase genes involved in defense response against oomycetes. Additionally, Novel-miR34, Novel-miR59, and tcc-miR156b were predicted to target cellulose synthase-like protein genes and expansin genes, which involved in cell wall synthesis and extension, an important stage in plant defense mechanisms against pathogen infection. Moreover, upregulated miRNAs, such as Tca-MIR3627a, target processes related to photosynthesis, including the activation of the RuBisCO protein. tcc-miR167a and Novel-miR6 target genes associated with protein ubiquitination, while Novel-miR18 targets processes involved in DNA damage checkpoint signaling and DNA repair.

In the case of downregulated miRNAs, a total of 1085 transcripts in *T. cacao* were predicted to be targeted. Among these, 207 disease-resistant genes were identified as targets for Novel-miR2, Novel-miR39, Novel-miR9, Tca-MIR472a, Tca-MIR482a, and Tca-MIRN9. Furthermore, several miRNAs were found to target 60 receptor protein kinase genes, which play a vital role in plant defense by recognizing pathogens. These miRNAs include Novel-miR2, Novel-miR39, Novel-miR9, Tca-MIR472a, Tca-MIRN9, and tcc-miR396c. Notably, Novel-miR9 was predicted to target receptor protein kinases containing L-type lectin-domains. In addition to targeting disease resistance genes, Novel-miR2 and Novel-miR9 were also predicted to target genes involved in cell wall structural components. According to the prediction, one particular miRNA, tcc-miR399a, is expected to target proteins that undergo the process of ubiquitination.

Up to 8917 *P. palmivora* genes were predicted to be controlled by 54 known miRNAs and 67 novel miRNAs, with 7245 genes were regulated through cleavage and 1672 genes through translational inhibition. Among the 9 upregulated miRNAs, several miRNAs exhibit multiple target genes in *P. palmivora* transcripts, where tcc-miR167a and Novel-miR18 are miRNAs with the highest number of target genes, up to 132 and 130 target genes, respectively then followed by Novel-miR6, Tca-MIR3627a, Novel-miR59, tcc-miR156b, Tca-MIR3954a, and Novel-miR34 with the number of target genes 97, 80, 76, 64, 63, and 52 respectively. However, Novel-miR42 was found to have no target genes.

The 9 upregulated miRNAs were predicted to regulate up to 694 genes in *P. palmivora*. Among these, 12 genes were RxLR effector genes, responsible for *P. palmivora* virulence factors that elicit plant hypersensitive response. Novel-miR59, Tca-MIR3627a, Novel-miR6, tcc-miR156b, and tcc-miR167a regulated these 12 genes. Moreover, Novel-miR6 and Novel-miR18 were also predicted to target Crinkler (CRN) family protein effector genes, which play a role in *P. palmivora* virulence factors by inducing necrosis in host plants. Other upregulated miRNAs were also predicted to target important genes in *P. palmivora*, including tcc-miR167a and Novel-miR18, which target cytochrome oxidase subunit genes involved in cellular respiration, Tca-MIR3627a targeting vesicle coat complex genes involved in intracellular protein transport, Novel-miR34, tcc-miR167a, Tca-MIR3954a, Novel-miR18, Novel-miR59, Novel-miR6 and tcc-miR156b targeting protein kinases involved in protein phosphorylation, and Tca-MIR3954a targeting transcription factor genes.

Meanwhile, the 8 downregulated miRNAs were predicted to target up to 621 genes. Among these genes, 8 were identified as RxLR effector genes, regulated by Novel-miR2, Tca-MIR482a, Tca-MIRN9, tcc-miR396c and tcc-miR399a, while 4 were identified as Crinkler family (CRN) effector genes, regulated by tcc-miR396c and Novel-miR39. Other downregulated miRNAs were also predicted to target important genes in *P. palmivora.* For instance, Tca-MIR482a which target cytochrome oxidase subunit genes, tcc-miR396c targeting pre-mRNA-splicing factor SYF1-like protein gene involved in pre-mRNA splicing, Tca-MIR472a targeting transmembrane protein gene, and Novel-miR39, tcc-miR396c, tcc-miR399a and Novel-miR9 targeting TKL/DRK protein kinase gene.

## Discussion

Cacao, belonging to the genus Theobroma, is a crop of significant economic importance^12^. Understanding the molecular defense mechanisms of cacao plants and their ability to suppress pathogen development is crucial in tackling black pod disease (BPD) caused by *P. palmivora*. Nonetheless, no previous analysis has compared the occurrence of miRNAs in regulating genes between resistant and susceptible species affected by BPD caused by *P. palmivora.* In this study, the identification of miRNAs that potentially target important mRNAs involved in plant defense and pathogen suppression was successfully conducted.

In the present study, we identified a total of 54 known miRNAs, consisting of 40 distinct miRNA families. In another study, Cheng et al.^13^ successfully identified 31 miRNA families in banana roots infected with *Fusarium oxysporum* f. sp. *Cubense.* Meanwhile, Rabuma et al.^7^ also succeeded in identifying 24 miRNA families in chili leaves infected with *Phytophthora capsici.* Xin et al.^14^ also conducted a study on miRNA identification of wheat infected with *Erysiphe graminis,* pathogen that causes powdery mildew disease in wheat, where the result showed that there were 132 miRNA families identified. The number of miRNA families involved in pathogenesis interaction is diverse and more studies are required to reveal key families.

Subsequently, we discovered a set of 67 miRNAs classified as novel, predicted by using MiRDeep2. In a similar study that employed the same program, Cheng et al.^13^ identified 28 novel miRNAs responsive to *Fusarium oxysporum* f. sp. *cubense* tropical race 4 (*Foc* TR4) in banana roots. However, only 5 novel miRNAs were found to have putative target genes. Another study by Liu et al.^16^ analyzed putative novel miRNAs and successfully identified 310 novel miRNAs associated with the ripening process of hot pepper (*Capsicum annum* L.). The discovery of putative novel miRNAs offers more candidate to explore to better understand post-transcriptional regulation in crucial events in plants.

In our study, the identified miRNAs exhibited a length range of 18–27 nucleotides, with the majority (60 miRNAs) having a length of 21 nucleotides. This finding agrees with Zhang et al.^17^ research on Peony flowers with identified miRNAs lengths ranging from 18 to 24 nucleotides. Guo et al.^18^ also identified soybean miRNAs with lengths varying from 18 to 25 nucleotides. Another study that aligns with these findings is Liu et al.^16^, which observed that miRNAs vary in length from 18 to 28 nucleotides during the stage of chili fruit ripening. Most miRNAs fall within the range of 20–24 nucleotides, with 24 nucleotides being the most abundant, additionally, 23 nucleotides are the typical length observed for miRNAs. Through numerous comparisons of miRNA lengths derived from numerous studies, it becomes evident that the length of miRNAs varies depending on the organism type and the specific role of miRNA in regulating processes within certain plants or growth phases.

We also predicted the pre-miRNAs structure of known and novel pre-miRNA with the highest abundance (Supplementary data, Fig. 1). pre-miRNAs must form a loop structure to be processed into mature miRNA. The stability of the pre-miRNA structure is determined by its MFE (minimum free energy) value, where a lower MFE indicates a more stable structure. MFEs of some of the most abundant miRNAs range from − 39.70 to − 25.60 kcal/mol for known miRNAs and from − 42.60 to − 14.30 kcal/mol for miRNAs that are novel. In another study, Tiwari et al.^19^ showed that the MFE of known and novel pre-miRNA of potato plant with low nitrogen stress were ranging between − 114.9 and − 19.9 kcal/mol for known miRNAs and − 166.4 and − 15.4 kcal/mol for novel miRNAs. The stability of pre-miRNA secondary structure will also determine the abundance of mature miRNAs in response to pathogen.

A study in chili pepper plant^22^ indicates that susceptibility could also arise from the regulation of gene expression, which is mediated by miRNAs suppressing their disease-resistance genes. We hypothesized that susceptibility in *Theobroma cacao* is also emphasized by miRNAs targeting resistance genes. The PsRNA analysis of the *T. cacao* genome identified 9 upregulated miRNAs predicted to target 1112 genes. tcc-miR156b has been identified as a potential regulator of the putative *T. cacao* gene that encodes the disease resistance RPP13-like protein through mRNA cleavage. Similarly, Novel-miR34 is predicted to target the same gene by inhibiting its translation at the mRNA level. Lewis et al.^23^ postulated in their research that the ZAR1 protein, which shares homology with RPP13, serves as a recognition factor for type III bacterial pathogen effectors (T3Es) like *Pseudomonas syringae*. ZAR1 belongs to the coiled-coil (CC) nucleotide-binding site and leucine-rich repeat (NBS-LRR) protein class, which includes the R protein.

Furthermore, tcc-miR167a, Novel-miR34, and Novel-miR6 were also predicted to target disease-resistance genes, specifically RGA, At4g19060, and At4g27220, through mRNA cleavage. The RGA (resistance gene analog) protein provides plant protection against pathogens by interacting directly or indirectly with avirulence proteins found in the pathogens. This interaction triggers a defense response that inhibits pathogen growth^24^. The proteins At4g19060 and At4g27220 belong to the NLR (nucleotide-binding, leucine-rich repeat) protein family, which detects pathogens presence and activity. NLR proteins can directly recognize pathogen effectors by binding to them through the LRR domain or indirectly detect modifications in host proteins caused by effector actions. In certain cases, host protein domains targeted by pathogen effectors have become integrated or fused into NLR proteins^25,26^.

In addition to their targeting of plant resistance genes, the four upregulated miRNAs, Tca-MIR3627a, Novel-miR6, Tca-MIR3954a, and Novel-miR34, are also predicted to target seven L-type lectin-domain containing receptor kinases. Wang et al.^27^ have reported the involvement of L-type lectin receptor kinases (LecRK) in the resistance response against oomycete pathogens such as *Phytophthora infestans* and *Phytophthora capsici.*

Several miRNAs, including Novel-miR34, Novel-miR6, Tca-MIR3954a, Novel-miR18, Novel-miR59, and tcc-miR156b, are predicted to target cellulose synthase-like genes and expansin genes, which are essential for cell wall synthesis and elongation. These processes play a crucial role in plant defense against pathogen infection. Specifically, cellulose synthase-like proteins are necessary for synthesizing cell wall polysaccharides required for root hair elongation in rice. They are considered functional orthologs of CSLD3/KOJAK in the Arabidopsis^28^. On the other hand, expansin genes contribute to the loosening and extension of plant cell walls by disrupting non-covalent bonds between cellulose microfibrils and the glucan matrix.

In addition to the upregulated miRNAs, the downregulated miRNAs identified in this study also exert significant regulatory roles in modulating the expression of various genes associated with plant defense against pathogens. Notably, seven downregulated miRNAs, namely Novel-miR2, Novel-miR39, Novel-miR9, Tca-MIR472a, Tca-MIR482a, Tca-MIRN9, tcc-miR399a, and tcc-miR396c, were predicted to target disease resistance genes, receptor protein kinases, and cell wall structural components. The downregulation of these miRNAs leads to decreased repression activity, enabling their target transcripts to be regulated normally without any specific treatment. This finding suggests that these miRNAs’ reduced expression levels may contribute to maintaining proper gene expression patterns associated with plant resistance mechanisms.

Identifying miRNAs that target their mRNA within the genome provides potential sources of plant resistance by eliminating specific miRNAs, leading to the continuous improvement of resistance genes. Silencing miRNAs using methods such as employing long noncoding RNA (lncRNA) represents an approach to weaken the repressive effects of miRNAs on their target genes^7,8^. For example, the elimination of miR482/2118s by lncRNAs acting in a ‘zig-zag-zig’ model of the plant immune system increases the levels of the target gene NBS-LRR which is an important component of ETI^29^.

Another strategy to inhibit miRNA expression and alleviate gene repression is the utilization of CRISPR/Cas9-based genome editing. In a study conducted by Hong et al.^30^, the MIR482 family, which targets the NBS-LRR protein, was successfully knocked out, resulting in enhanced plant resistance to *Phytophthora infestans* in tomato plants. Interestingly, the study also observed a decrease in reactive oxygen species (ROS) activity in response to plant stress. These findings are consistent with previous report by Hong et al.^31^, which demonstrated that upregulation of MIR482c in tomato led to an increase in ROS levels and a decrease in the expression of POD and SOD coding genes, indicating the involvement of miRNA-mediated regulation in plant stress responses.

Another form of plant defense against pathogen infection is cross kingdom RNAi. This defense mechanism involves the transfer of small regulatory RNA molecules into the invading pathogen, subsequently initiating the silencing of virulence genes within the pathogen genome^32^. The silencing of these virulence genes critically undermines the pathogen ability to establish infection, thereby suppressing infection^32^.

Schaefer et al.^33^ showed that suppression of *SvrPm3a1/f1, Bgt_Bcg-6*, and *Bgt_Bcg-7* effectors in wheat with RNAi reduced effector expression and increase plant resistance to infection of powdery mildew. Wang et al.^34^ also found that Botrytis could uptake exogenous sRNA which in this study is an sRNA specifically targeting the *Bc-DCL1* and *Bc-DCL2* genes involved in synthesizing pathogenic effectors. The uptake then caused repression of effector production thereby suppressing infection by the pathogen.

Here, we also found the possible cross kingdom RNAi interaction that *T. cacao* miRNA targets mRNA of *P. palmivora.* Target analysis on *P. palmivora* transcripts revealed that 9 upregulated miRNAs were predicted to regulate 694 genes. Among them, 12 were identified as RxLR effector genes, which are regulated by Novel-miR59, Tca-MIR3627a, Novel-miR6, tcc-miR156b, and tcc-miR167a. RxLR effectors represent a group of proteins characterized by RxLR (Arginine-any amino acid-leucine-arginine) motifs highly conserved at the N-terminal while displaying greater diversity at the C-terminal region. The N-terminal segment facilitates the translocation of the effector into the host cell, whereas the C-terminal region is involved in the effector's activity and function^36^. Pathogens release effectors to counter the plant PTI response. Within the host cell, RxLR effectors will target diverse components within the plant defense system. This could be achieved by disrupting signal transduction pathways or modifying specific proteins functionality^37^. Upon recognition by host R proteins, RxLR proteins elicit host effector-triggered immunity (ETI) by triggering a hypersensitive response (HR).

Novel-miR6 and Novel-miR18 are also predicted to target Crinkler (CRN) family protein effector genes. CRN effectors are characterized by an N terminus containing a highly conserved LxLFLAK (leucine-any amino acid-phenylalanine-leucine-alanine-lysine) motif, while their C terminus exhibits more sequence diversity. These CRN effectors mainly induce necrosis in host cells^38,39^. Silencing of these effector genes can suppress *P. palmivora* infection. Hou et al.^40^ demonstrated that targeting effector PSR2 of *Phytophthora capsici* using siRNA can effectively suppress the infection of *P. capsici* in *Arabidopsis thaliana.*

Other miRNAs were also predicted to target important genes in *P. palmivora*, including genes involved in respiration, glycolysis, protein transport, protein phosphorylation, transcription factors, transmembrane proteins and mRNA processing. These genes play critical roles in various metabolic activities of *P. palmivora*. Silencing these genes can disrupt the growth of *P. palmivora*. The study conducted by Hu et al.^41^ demonstrated that targeting genes encoding acetyl-CoA, 40S ribosomal protein, and glycine cleavage system in *Phakopsora pachyrhizi* using dsRNA can effectively suppress the growth of *P. pachyrhizi*.

miRNAs possess notable environmentally friendly characteristics and exhibit high specificity towards targets, making them a promising candidate to be used in developing plant protection methods with the RNAi approach^42^. Currently, many RNAi approaches rely on transgenic plants expressing double-stranded RNA^43^, However, public reaction towards transgenic plants has been unfavorable. Koch et al.^11^ demonstrated that by applying CYP3-dsRNA to target *Fusarium graminearum CYP3* gene through spraying or Spray-Induced Gene Silencing (SIGS) effectively repressed the infection of *F. graminearum* on barley. This finding highlights the potential of SIGS as an environmentally friendly alternative for plant protection methods. Therefore, identifying miRNAs that target pathogen effectors provides potential sources of plant resistance by repressing the expression of effector genes, thereby suppressing infection and improving plant resistance against pathogens.

In general, this study also holds the potential in contributing to miRNA-based breeding strategies aimed at improving crop traits. miRNAs are known to play a role in various functions in plants, including response to stress, disease resistance regulation, flower development, and grain yield^44^. Identification and elimination of disease- and pathogen-associated miRNAs, as well as identification and overexpression of miRNAs associated with crop yield and quality, can serve as effective strategies in molecular breeding. Hong et al.^30^ showed that elimination of miR482b and miR482c, which are known to contribute to tomato susceptibility to *P. infestans,* resulted in increased resistance of tomato plants to *P. infestans* infection. Zhang et al.^45^ also showed that overexpression of miRNA OsmiR397 could increase grain size and promote panicle branching, hence promoting rice yield.

Additionally, miRNAs can also serve as molecular markers in plant breeding programs. Due to their tissue and developmentally-specifics expression patterns, miRNAs can be used as reliable markers for trait selection and identification of elite germplasms. Sihag et al.^46^ succeeded in developing heat-responsive miRNA-SSE markers for the characterization of wheat germplasm for terminal heat tolerance breeding. Mondal et al.^47^ also successfully identified salt responsive miRNA-SSR markers in rice. The development of miRNA-based molecular markers allows for efficient and precise selection of desired traits, reducing the time and resources required for traditional breeding methods.

In our study, we also identified seven promising miRNAs (Table 4) with potential for further research and development. Among them, five miRNAs, namely Tca-MIR3954a, Novel-miR34, tcc-miR396c, Tca-MIR472a, and Tca-MIR482a, were identified as targets for elimination in *T. cacao* transcripts. These miRNAs play important roles in pathogen resistance and cell wall formation in cacao plants. By eliminating the suppressive effects on their target genes, it is expected that deletion of these five miRNAs in cacao plants will improve their resistance to pathogens. In addition, two miRNAs, Novel-miR6 and tcc-miR399a, were identified as potential candidates for biocontrol agents against *P. palmivora*. These miRNAs are predicted to target effector proteins in the pathogen. By applying these synthetic miRNAs to cacao plants, the expression of effector genes in *P. palmivora* can be targeted and repressed, thus suppressing the infection caused by *P. palmivora* in cacao plants.

The selection of these seven miRNAs from a total of 17 differentially expressed miRNAs was based on their targets in *T. cacao* transcripts and genes in *P. palmivora* that are not contradictory in enhancing resistance. This discovery potentially advances our understanding of the molecular mechanisms at the miRNA level in the interactions between cacao plants and the pathogen *P. palmivora*.

## Conclusion

In conclusion, identifying and characterizing miRNAs in cacao plants provide valuable insights into the mechanisms governing plant defense against black pod disease caused by *P. palmivora* in infected cacao fruits compared to healthy ones. Our study identified five miRNAs, Tca-MIR3954a, Novel-miR34, tcc-miR396c, Tca-MIR472a, and Tca-MIR482a with promising potential for future engineering, such as CRISPR/Cas9 or LncRNA methods, to suppress the expression of miRNAs targeting their own resistance genes during pathogen response. Additionally, we propose synthesizing two miRNAs, Novel-miR6 and tcc-miR399a, that can be utilized as biocontrol agents to suppress the expression of pathogenesis-related mRNAs in *P. palmivora*.

## Materials and methods

### Plant material and sample preparation

The cacao samples were obtained from the Indonesian coffee and cocoa research institute plantation in Jember, East Java, Indonesia. Two varieties, namely TSH858 (resistant, healthy) and Sulawesi 1 (susceptible, infected), were selected for the study. Each variety was collected in two technical replicates, labeled as R1 and R2 for the resistant samples, and S1 and S2 for the susceptible samples.

Disease scoring was performed on two samples, R and S, by measuring the length and width of the pod. Apart from that, the transverse diameter 1 and longitudinal diameter 2 of the rotten fruit were also measured. Diameter measurements are used to determine area and growth rate^20^. The diseased area (cm^2^) was calculated based on Eq. (1).

1 $$A = \left( {\frac{{d_{1} + d_{2} }}{4}} \right)^{2} \times \pi$$

The obtained measurements were then used in the cacao pod rot area formula to calculate the average and determine the disease score or the resistance parameter of the cacao pod against *P. palmivora*^48^. Following the disease scoring, the cacao pod samples were thinly sliced and freeze-dried using liquid nitrogen to preserve the integrity of the samples. Afterward, the samples were stored at − 80 °C until further analysis.

The presence of *P. palmivora* on cacao was confirmed through the amplification of the ITS2 and 5.8S rDNA genes of *P. palmivora*. The specific primers utilized for ITS2 amplification were designed based on the accession number KU172528.1. The forward primer sequence was 5′–TTCGCGGTATGGTTGGCTTC–3′, while the reverse primer sequence was 5′–GCCGAAGCATACACAAGTTCC–3′. The selection of the 5.8S rDNA primer was based on the research conducted by Darmono et al.^49^ where the forward primer employed was 5′–CTTAGTTGGGGGTCTCTTTC–3′ and the reverse primer was 5′–GTTCACCAATCATACCACC–3′. The PCR process involved the following steps: preheating the sample to 95 °C for 3 min and denaturation at 95 °C for 15 s. The annealing step was performed at 59 °C for *ITS2* and 54 °C for 5.8S *rDNA*, each for 15 s. Elongation of the DNA strands occurred at 72 °C for 10 s, followed by a final elongation at 72 °C for 3 min. The amplified products were then sequenced to confirm the presence of *P. palmivora.*

### Small RNA extraction and illumina sequencing

Total small RNA was extracted using mirPremier® microRNA Isolation Kit by Sigma-Aldrich, Merck, Germany, per manufacture instruction. The isolates were measured for purity and concentration using a nanodrop spectrophotometer (ThermoFisher Scientific, USA) and band quality was visualized using a 4% agarose gel. Total small RNAs from four samples were then sequenced on the Illumina Novoseq 6000 platform (Novogene, Singapore) and library preparation followed the guidelines the manufacturers provided. Each sample was sequenced individually. After passing the library preparation quality assessment, the library was sequenced on the illumina Novoseq 6000 platform with a read length of 50 bp.

### Reads quality check and filtering

The output of small RNA sequencing was checked for quality reads using the FastQC v0.11.9^50^. The presence of contaminating adapters and overrepresented sequences was eliminated using the Trimmomatic v0.39^51^. Then the reads were filtered to obtain miRNAs with a minimum length of 18 bp and a maximum of 28 bp. The targeted miRNA length was then subjected to further analysis.

### Small RNA annotation and miRNA identification

After removing the low-quality reads that contain 5′ adapter contaminants and overrepresented sequences from raw data, the clean reads obtained were mapped to reference the cacao genome using Rsubread v2.10.5^52^. For known miRNAs, reads were quantified using feature counts from Rsubread based on two cacao miRNA databases, PmiREN (https://www.pmiren.com/) and miRBase (https://www.mirbase.org/). The remaining unannotated reads were then subjected to novel miRNA prediction using MiRDeep2 v0.1.3^15^. The predicted miRNA obtained was then quantified using feature counts from Rsubread. pre-miRNAs structure and their minimum free energy (MFE) were also predicted with RNAfold^53^.

### Differential expression analysis and target prediction

The known and novel miRNA quantification results were then analyzed for differential expression using the Deseq2 v1.36.0^54^. A fold change > 1 with *p*adj < 0.05 was considered as differentially expressed miRNAs (DE miRNA) that were upregulated, and fold change < 1 with *p*adj < 0.05 was considered as DE miRNAs that were downregulated. DE miRNAs were then visualized in EnhancedVolcano v1.14.0^55^ and heatmap v1.0.12^56^. The target gene of significantly upregulated miRNAs in the cacao and *P. palmivora* genomes were then predicted using psRNATarget v2^57^, BLAST^48^ and uniport^35^. Prior to BLAST, miRNA sequences were reverse complemented using FastX Toolkit program^21^. This process converts the miRNA sequences into their corresponding target sequences.

### Supplementary Information

Supplementary Information.

## Data Availability

The miRNA sequencing data of 4 samples reported in this study have been deposited into the NCBI Geo Expression Omnibus with GEO accession GSE244955 that are publicly accessible at https://www.ncbi.nlm.nih.gov/geo/query/acc.cgi?acc=GSE244955.
