# Supplementary material for: Identification of self- and pathogen-targeted miRNAs from resistant and susceptible Theobroma cacao variety to black pod disease
Source: Sci Rep. 2024 Feb 8;14:3272. doi: 10.1038/s41598-024-53685-x (PMC10853554; doi:10.1038/s41598-024-53685-x)
Supplement: Supplementary file 1 — Supplementary Information. [file 41598_2024_53685_MOESM1_ESM.docx]

**Supplementary Information**

**Supplementary Table 1.** Confirmation the presence of *P. palmivora*

| **Sampel** | **PCR amplicon** | | **BLAST result** | |
| --- | --- | --- | --- | --- |
|  | **ITS2** | **5.8S rDNA** | **ITS2** | **5.8S rDNA** |
| Resistance | - | - | - | - |
| Susceptible | + | + | *P. palmivora* *ITS*2 partial sequence (MF370569.1),  *Query cover*: 92%  *Percent identity:* 89% | *P. palmivora* 5.8S ribosomal RNA complete sequence (KR818196.1),  *Query cover*: 97%  *Percent identity*: 99,83% |

**Supplementary Table 2.** Results of small RNA sequencing and analysis in resistance (R1 & R2) and susceptible (S1 & S2) libraries of *T. cacao*

|  | **Sample** | | | |
| --- | --- | --- | --- | --- |
|  | **R1** | **R2** | **S1** | **S2** |
| **Total reads** | 12,251,966 | 13,400,032 | 11,678,902 | 9,679,773 |
| **Reads with low quality** | 0 | 0 | 0 | 0 |
| **Reads with N% > 10%** | 0 | 0 | 0 | 701 |
| **Clean reads** | 11,124,113 | 2,974,499 | 5,638,987 | 5,306,651 |
| **18–28 nt sRNAs** | 10,685,251 | 2,856,473 | 3,861,549 | 4,134,082 |
| **Mapped reads** | 3,999,744 | 1,619,130 | 1,218,792 | 1,192,304 |

**Supplementary Table 3.** List of miRNAs and their abundance

| **miRNA ID** | **Sequence (5'-3')** | **Chromosome** | **Start position** | **End position** | **Strand** | **R1** | **R2** | **S1** | **S2** |
| --- | --- | --- | --- | --- | --- | --- | --- | --- | --- |
| tcc-miR156a | TGACAGAAGAGAGAGAGCACA | NC_023624.1 | 40422509 | 40422529 | - | 0 | 0 | 0 | 0 |
| tcc-miR156b | TGACAGAAGAGAGTGAGCAC | NC_023617.1 | 1627358 | 1627377 | - | 65 | 17 | 9 | 15 |
| tcc-miR156e | TTGACAGAAGATAGAGAGCAC | NC_023622.1 | 1544555 | 1544575 | - | 26 | 1 | 2 | 2 |
| tcc-miR160a | TGCCTGGCTCCCTGAATGCCA | NC_023617.1 | 34637581 | 34637601 | - | 174 | 107 | 8 | 13 |
| tcc-miR160b | TGCCTGGCTCCCTGTATGCCA | NC_023621.1 | 2805543 | 2805563 | - | 27 | 3 | 0 | 2 |
| tcc-miR160c | TGCCTGGCTCCCTGGATGCCA | NC_023625.1 | 14143990 | 14144010 | - | 0 | 1 | 0 | 0 |
| tcc-miR162 | TCGATAAACCTCTGCATCCAG | NC_023623.1 | 22612635 | 22612655 | - | 1260 | 754 | 23 | 18 |
| tcc-miR164a | TGGAGAAGCAGGGCACGTGCA | NC_023621.1 | 228336 | 228356 | - | 183 | 11 | 2 | 0 |
| tcc-miR164c | TGGAGAAGCAGGGCACATGCT | NC_023620.1 | 21296396 | 21296415 | + | 0 | 0 | 0 | 0 |
| tcc-miR166a | TCGGACCAGGCTTCATTCCCC | NC_023623.1 | 28905616 | 28905636 | - | 2302 | 183 | 20 | 30 |
| tcc-miR166c | TCGGACCAGGCTTCATTCCTC | NC_023625.1 | 8316424 | 8316444 | - | 9 | 13 | 0 | 1 |
| tcc-miR167a | TGAAGCTGCCAGCATGATCTA | NC_023621.1 | 37920904 | 37920924 | - | 57 | 2 | 3 | 10 |
| tcc-miR169a | CAGCCAAGGATGACTTGCCGA | NC_023621.1 | 36120254 | 36120274 | + | 1 | 0 | 0 | 0 |
| tcc-miR169b | CAGCCAAGGATGACTTGCCGG | NC_023624.1 | 41248076 | 41248096 | - | 0 | 0 | 0 | 0 |
| tcc-miR169d | TAGCCAAGGATGACTTGCCTA | NC_023624.1 | 13634890 | 13634909 | - | 0 | 0 | 0 | 0 |
| tcc-miR169f | AAGCCAAGAATGACTTGCCTG | NC_023617.1 | 35685997 | 35686017 | - | 0 | 0 | 0 | 0 |
| tcc-miR169h | TAGCCAAGGATGACTTGCCTG | NC_023624.1 | 13634889 | 13634909 | - | 0 | 0 | 0 | 0 |
| tcc-miR169m | TGAGCCAAGGATGACTTGCCG | NC_023624.1 | 41248077 | 41248095 | - | 0 | 0 | 0 | 0 |
| tcc-miR171a | TGATTGAGCCGCGCCAATATC | NC_023625.1 | 37241394 | 37241414 | - | 0 | 0 | 0 | 0 |
| tcc-miR171d | TGATTGAGCCGTGCCAATATC | NC_023620.1 | 26335428 | 26335448 | - | 31 | 2 | 0 | 0 |
| tcc-miR172a | GGAATCTTGATGATGCTGCA | NC_023617.1 | 39564261 | 39564280 | - | 0 | 0 | 0 | 0 |
| tcc-miR172b | AGAATCTTGATGATGCTGCAT | NC_023617.1 | 41124949 | 41124969 | - | 40 | 1 | 1 | 0 |
| tcc-miR172c | GGAATCTTGATGATGCTGCAT | NC_023617.1 | 39564260 | 39564280 | - | 0 | 0 | 0 | 0 |
| tcc-miR172d | AGAATCCTGATGATGCTGCAT | NC_023621.1 | 3119471 | 3119491 | - | 101 | 12 | 0 | 0 |
| tcc-miR2111 | TAATCTGCATCCTGAGGTTTA | NC_023622.1 | 2249987 | 2250005 | - | 0 | 0 | 0 | 1 |
| tcc-miR390a | AAGCTCAGGAGGGATAGCGCC | NC_023621.1 | 387538 | 387558 | - | 148 | 25 | 2 | 2 |
| tcc-miR393a | TCCAAAGGGATCGCATTGATCC | NC_023616.1 | 1493699 | 1493720 | - | 0 | 0 | 0 | 0 |
| tcc-miR393b | TCCAAAGGGATCGCATTGATC | NC_023623.1 | 26617403 | 26617423 | - | 14 | 0 | 0 | 0 |
| tcc-miR395a | CTGAAGTGTTTGGGGGAACTC | NC_023624.1 | 41716786 | 41716806 | - | 1117 | 225 | 16 | 27 |
| tcc-miR396a | TTCCACAGCTTTCTTGAACTG | NC_023617.1 | 7261338 | 7261358 | - | 591 | 53 | 15 | 15 |
| tcc-miR396c | TTCCACAGCTTTCTTGAACTT | NC_023620.1 | 2612756 | 2612776 | - | 2392 | 233 | 3 | 5 |
| tcc-miR396d | TTCCACGGCTTTCTTGAACTT | NC_023624.1 | 8719738 | 8719758 | - | 48 | 8 | 2 | 0 |
| tcc-miR397 | TCATTGAGTGCAGCGTTGATG | NC_023620.1 | 25408012 | 25408032 | - | 35 | 6 | 0 | 0 |
| tcc-miR399a | CGCCAAAGGAGAGTTGCCCTG | NC_023623.1 | 31602471 | 31602491 | - | 915 | 586 | 1 | 0 |
| tcc-miR399b | TGCCAAAGGAGATTTGCCCGG | NC_023620.1 | 24721106 | 24721124 | - | 0 | 0 | 0 | 0 |
| tcc-miR399e | CGCCAAAGGAGAATTGCCCTG | NC_023625.1 | 9344749 | 9344769 | - | 0 | 0 | 0 | 0 |
| tcc-miR399g | TGCCAAAGGAGAATTGCCCTG | NC_023625.1 | 9353697 | 9353717 | - | 3 | 0 | 0 | 0 |
| tcc-miR399h | TGCCAAAGGAGATTTGCCCCG | NC_023625.1 | 9349958 | 9349978 | - | 0 | 0 | 0 | 0 |
| tcc-miR399i | TGCCAAAGGAGAGTTGCCCTG | NC_023623.1 | 25998277 | 25998297 | - | 156 | 18 | 0 | 2 |
| tcc-miR403a | TTAGATTCACGCACAAACTCG | NC_023622.1 | 32849355 | 32849375 | - | 3260 | 212 | 3 | 16 |
| tcc-miR535 | TGACAACGATAGAGAGCACGC | NC_023625.1 | 15365379 | 15365399 | - | 769 | 97 | 5 | 13 |
| tcc-miR827 | TTAGATGACCATCAACAAACA | NC_023621.1 | 101440 | 101458 | - | 76 | 3 | 0 | 1 |
| Tca-MIR159a | ATTGGACTGAAGGGAGCTCC | NC_023623.1 | 2319894 | 2319913 | + | 11 | 3 | 0 | 1 |
| Tca-MIR159b | TTGGACTGAAGGGAGCTCCC | NC_023623.1 | 32655000 | 32655019 | - | 687 | 111 | 21 | 61 |
| Tca-MIR159d | TTGGACTGAAGGGAGCTCCT | NC_023616.1 | 25272801 | 25272821 | + | 48938 | 22272 | 357 | 528 |
| Tca-MIR165a | TTGGACCAGGCTTCATTCCCC | NC_023623.1 | 28184064 | 28184084 | + | 1381 | 61 | 2 | 4 |
| Tca-MIR165g | TCTCGGACCAGGCTTCATTCC | NC_023622.1 | 30265368 | 30265388 | - | 64 | 22 | 2 | 0 |
| Tca-MIR170a | TTGAGCCGCGCCAATATCACT | NC_023620.1 | 2319830 | 2319850 | + | 0 | 0 | 0 | 0 |
| Tca-MIR170b | TATTGGCCTGGTTCACTCAGA | NC_023625.1 | 481611 | 481631 | - | 0 | 3 | 0 | 0 |
| Tca-MIR3627a | TTGTCGCAGGAGCGATGGCACT | NC_023622.1 | 13565389 | 13565410 | + | 7 | 0 | 4 | 6 |
| Tca-MIR394a | TTGGCATTCTGTCCACCTCC | NC_023624.1 | 2087639 | 2087658 | + | 23 | 5 | 0 | 0 |
| Tca-MIR3954a | TTGGACAGAGTAATCACGGTCG | NC_023622.1 | 30293905 | 30293926 | - | 2992 | 182 | 111 | 311 |
| Tca-MIR398a | TGTGTTCTCAGGTCGCCCCTG | NC_023622.1 | 26691506 | 26691526 | + | 128 | 81 | 10 | 1 |
| Tca-MIR399a | CGCCAAAGGAGAGTTGCCCTG | NC_023623.1 | 31602471 | 31602491 | - | 0 | 0 | 0 | 0 |
| Tca-MIR408a | ATGCACTGCCTCTTCCCTGGC | NC_023625.1 | 34189658 | 34189678 | - | 711 | 94 | 7 | 0 |
| Tca-MIR472a | TCTTCCCTACTCCACCCATCCC | NC_023621.1 | 40294561 | 40294582 | - | 6525 | 490 | 3 | 21 |
| Tca-MIR477a | TGAAGTCTTTGGGGAGAGTGA | NC_023623.1 | 32904070 | 32904090 | + | 22 | 4 | 0 | 1 |
| Tca-MIR477b | ACTCTCCCTCAAGGGCTTCAC | NC_023624.1 | 8461375 | 8461395 | + | 42 | 8 | 0 | 0 |
| Tca-MIR482a | TCTTCCCAACACCTCCCATACC | NC_023624.1 | 22810014 | 22810035 | + | 20440 | 950 | 8 | 20 |
| Tca-MIR5225a | CTGTCGGAGGAGAGATAGCGCC | NC_023622.1 | 13568569 | 13568590 | + | 46 | 6 | 0 | 1 |
| Tca-MIR530a | TGCATTTGCACCTGCACCTTC | NC_023617.1 | 41222161 | 41222181 | + | 10 | 6 | 0 | 0 |
| Tca-MIR530b | TGCATTTGCACCTGCACCTCT | NC_023621.1 | 3339783 | 3339803 | + | 2 | 0 | 0 | 0 |
| Tca-MIRN1 | GAAGCGCTTGGGGAAGAGTGG | NC_023623.1 | 32904264 | 32904284 | + | 4 | 1 | 0 | 0 |
| Tca-MIRN10 | GGGGAACTGGACTCATAATCC | NC_023616.1 | 18797660 | 18797680 | - | 133 | 18 | 0 | 3 |
| Tca-MIRN2 | TGAAGTCCTTGGGGCGAGTGC | NC_023624.1 | 8336993 | 8337013 | + | 19 | 1 | 0 | 0 |
| Tca-MIRN3 | TTGCCCACGTGACATATGACT | NC_023617.1 | 34010113 | 34010133 | + | 88 | 6 | 0 | 0 |
| Tca-MIRN5 | TGTCTAGACAGAGAAACAGCAG | NC_023618.1 | 8712476 | 8712497 | + | 1016 | 34 | 3 | 2 |
| Tca-MIRN6 | CTACGATGTTGGCTCGGTTCA | NC_023618.1 | 2407001 | 2407021 | - | 0 | 0 | 0 | 0 |
| Tca-MIRN7 | AGGAACCTGAACCGAGAGTTC | NC_023619.1 | 19704238 | 19704258 | - | 154 | 31 | 0 | 2 |
| Tca-MIRN7068a | ACTCTCTTTCGAAGGCTTCAAG | NC_023625.1 | 35431242 | 35431263 | - | 5592 | 445 | 2 | 24 |
| Tca-MIRN7069a | GAGCTCTCTGAACTCCAAACC | NC_023625.1 | 35637711 | 35637731 | - | 0 | 0 | 0 | 0 |
| Tca-MIRN9 | TTCACTTCTCTGATTTGGGCGA | NC_023619.1 | 19178360 | 19178381 | - | 9948 | 341 | 2 | 19 |
| Novel-miR1 | TTGCCGAGTCCGCCCATGCCTA | NC_023624.1 | 22796221 | 22796242 | + | 31310 | 2312 | 84 | 128 |
| Novel-miR2 | TTGCCGGCTCCGCCCATTCCAA | NC_023624.1 | 22809818 | 22809839 | + | 3627 | 117 | 0 | 3 |
| Novel-miR3 | TCGGACCAGGCTTCATTCCCC | NC_023625.1 | 29987927 | 29987948 | + | 216 | 36 | 0 | 3 |
| Novel-miR4 | ACAGGGAACAGGCTGAGCATG | NC_023625.1 | 34189717 | 34189737 | - | 1180 | 107 | 13 | 8 |
| Novel-miR5 | CTTTTAACCGACTTTTGCACTCCC | NC_023617.1 | 4348336 | 4348356 | - | 1914 | 285 | 9 | 10 |
| Novel-miR6 | GATAAATCATTGCAATTGTTG | NC_023619.1 | 21211154 | 21211177 | + | 520 | 202 | 305 | 322 |
| Novel-miR7 | CTGAAGTGTTTGGGGGAACTC | NC_023621.1 | 2095642 | 2095662 | + | 105 | 34 | 0 | 0 |
| Novel-miR8 | TCGGTTATTCGGTCGGTTCGGGGG | NC_023621.1 | 34208570 | 34208593 | - | 506 | 25 | 2 | 1 |
| Novel-miR9 | TTGTCAACGATCTAGCTCAAT | NC_023616.1 | 24331034 | 24331054 | - | 228 | 91 | 0 | 0 |
| Novel-miR10 | TTGCTGTGGCCGGTATGCTGGATC | NC_023617.1 | 34837227 | 34837250 | + | 188 | 8 | 0 | 0 |
| Novel-miR11 | TTAAACGTGTTTGTCGTATCTGAC | NC_023623.1 | 26028974 | 26028997 | - | 85 | 4 | 0 | 2 |
| Novel-miR12 | TGGATCATGTGGTAGCTTCACC | NC_023625.1 | 30570919 | 30570942 | - | 101 | 21 | 0 | 0 |
| Novel-miR13 | TTACACATGGAAACAACGGTTG | NC_023618.1 | 8712332 | 8712353 | + | 125 | 2 | 1 | 0 |
| Novel-miR14 | ACCGATCGAGGCTCCCTGAGAGAGC | NC_023621.1 | 21746233 | 21746258 | - | 127 | 22 | 0 | 0 |
| Novel-miR15 | ACTCTCCCTCAAAGGCTTCAGG | NC_023623.1 | 32904020 | 32904041 | + | 84 | 8 | 0 | 0 |
| Novel-miR16 | ACATCGATCTAGTCATTTCTGACA | NC_023618.1 | 3862178 | 3862201 | + | 76 | 29 | 0 | 2 |
| Novel-miR17 | CGTGATATTGGTTCGGCTCATC | NC_023623.1 | 28100761 | 28100782 | - | 51 | 2 | 0 | 2 |
| Novel-miR18 | GCGGCGATGATGATTGAAAA | NC_023618.1 | 883902 | 883920 | + | 55 | 10 | 34 | 50 |
| Novel-miR19 | TAATTGTAGTAGACAACTAGACGT | NC_023616.1 | 23774235 | 23774258 | + | 40 | 10 | 0 | 1 |
| Novel-miR20 | ATGTACCGGTCACTTGTTCTTAGT | NC_023621.1 | 39261508 | 39261531 | + | 93 | 17 | 0 | 0 |
| Novel-miR21 | AGCTGCCGACTCATTCATTCA | NC_023617.1 | 41926678 | 41926698 | - | 31 | 3 | 0 | 0 |
| Novel-miR22 | CCGATTTCATCTTTTAATTCTGCAG | NC_023622.1 | 25077020 | 25077044 | + | 22 | 3 | 0 | 0 |
| Novel-miR23 | TATTCATCAGTTCGGTTGCTC | NC_023619.1 | 4240119 | 4240139 | - | 165 | 82 | 1 | 3 |
| Novel-miR24 | ACTCTCCCTCAAGGGCTTCCG | NC_023623.1 | 32904217 | 32904238 | + | 3 | 3 | 0 | 0 |
| Novel-miR25 | GATGATGTGGCAACATTGACGTGG | NC_023618.1 | 10532221 | 10532243 | - | 8 | 5 | 0 | 0 |
| Novel-miR26 | CAAGCATTGAAACTATCACAA | NC_023624.1 | 5660471 | 5660491 | - | 63 | 1 | 0 | 1 |
| Novel-miR27 | TAGATCTAGTAGGGAAACGCC | NC_023619.1 | 8138645 | 8138665 | + | 62 | 14 | 0 | 0 |
| Novel-miR28 | CGACTTAAAGATGAAGAAATA | NC_023621.1 | 663435 | 663455 | + | 5 | 1 | 1 | 0 |
| Novel-miR29 | TATGTGGCTTTGCATGATACT | NC_023620.1 | 24939686 | 24939706 | - | 34 | 3 | 0 | 5 |
| Novel-miR30 | TTCCATCTCTTGCACACTGGA | NC_023625.1 | 2196271 | 2196294 | - | 100 | 6 | 2 | 1 |
| Novel-miR31 | TGACAGAAGAGAGTGAGCACA | NC_023617.1 | 851039 | 851059 | + | 7 | 9 | 0 | 0 |
| Novel-miR32 | CAACGCTTGGAACACTTTTGGG | NC_023620.1 | 1990452 | 1990473 | + | 224 | 5 | 0 | 0 |
| Novel-miR33 | CAAGTTTCTCTGACCCACTCA | NC_023624.1 | 39888838 | 39888858 | + | 93 | 6 | 0 | 0 |
| Novel-miR34 | AATGTAAATCTGAACAATTC | NC_023619.1 | 572382 | 572402 | + | 27 | 8 | 5 | 20 |
| Novel-miR35 | ATAATCAGATCTGGCCATGGAGCA | NC_023619.1 | 4657319 | 4657342 | - | 25 | 4 | 0 | 0 |
| Novel-miR36 | CAAGGCTTTGAAATACAA | NC_023622.1 | 17016102 | 17016119 | - | 66 | 10 | 0 | 0 |
| Novel-miR37 | TGGCGATCTGGATTTGGTTCA | NC_023620.1 | 23727908 | 23727928 | - | 13 | 2 | 2 | 0 |
| Novel-miR38 | ATTTTAATTGGGAGGAAGGACATT | NC_023621.1 | 18422890 | 18422913 | - | 28 | 4 | 0 | 0 |
| Novel-miR39 | AGCTCCTGAGATATGCAAGC | NC_023623.1 | 4147363 | 4147383 | - | 816 | 70 | 0 | 0 |
| Novel-miR40 | ATGAGGATCTAGTCTGAAAGCACC | NC_023623.1 | 30123114 | 30123137 | + | 26 | 4 | 0 | 0 |
| Novel-miR41 | CACGTCGGTGCCACGTGGCATGCC | NC_023619.1 | 24287624 | 24287647 | + | 11 | 1 | 0 | 1 |
| Novel-miR42 | GATAACATCATAGGATTA | NC_023622.1 | 29085332 | 29085349 | + | 788 | 100 | 49 | 237 |
| Novel-miR43 | AATTCAAGTCTGGTAGAACATGCC | NC_023625.1 | 10434020 | 10434043 | + | 22 | 2 | 0 | 1 |
| Novel-miR44 | CTTACGACTAATGATTGACAAAT | NC_023623.1 | 32678929 | 32678951 | + | 307 | 34 | 3 | 2 |
| Novel-miR45 | AATTGGCAAAAGGAGAGGCTG | NC_023621.1 | 34386172 | 34386192 | + | 183 | 12 | 0 | 0 |
| Novel-miR46 | TCCTGATTCTAGAACCAAGACCT | NC_023616.1 | 14126373 | 14126395 | - | 13 | 3 | 0 | 0 |
| Novel-miR47 | AAGGAATTGGATTTTGAGAGAA | NC_023617.1 | 4845161 | 4845181 | + | 840 | 5 | 1 | 0 |
| Novel-miR48 | AAACGTGTTACTTGTGTCTGACA | NC_023618.1 | 19652016 | 19652038 | + | 162 | 60 | 0 | 1 |
| Novel-miR49 | CCTTGACGTGTAACATCTGATGTT | NC_023621.1 | 6101432 | 6101455 | - | 71 | 7 | 0 | 0 |
| Novel-miR50 | TTCCCGAAAAACTTGTAGAGACG | NC_023620.1 | 22203277 | 22203298 | + | 37 | 3 | 0 | 0 |
| Novel-miR51 | GTTTTGTGTCTAATCGTATCTGAT | NC_023622.1 | 24226531 | 24226554 | + | 107 | 9 | 3 | 1 |
| Novel-miR52 | ACTTCAATCTTCTACTTGTGTGCT | NC_023619.1 | 21843146 | 21843169 | - | 104 | 16 | 0 | 0 |
| Novel-miR53 | GTGTCTATAGAAATACTCTCATGT | NC_023617.1 | 36081059 | 36081082 | + | 16 | 4 | 0 | 0 |
| Novel-miR54 | GATACTGACGTGTGAAATGTGAAA | NC_023616.1 | 3857724 | 3857747 | - | 20 | 8 | 0 | 0 |
| Novel-miR55 | TTAAACGTGTTATTTGTGTCTGAC | NC_023622.1 | 6055064 | 6055087 | + | 93 | 7 | 2 | 1 |
| Novel-miR56 | TTCTTGAACTAACCTAAGGACTTC | NC_023623.1 | 704491 | 704514 | - | 34 | 7 | 0 | 0 |
| Novel-miR57 | GTTTCGTGTCTAATCGTGTCTGAT | NC_023618.1 | 9151686 | 9151709 | - | 38 | 4 | 0 | 2 |
| Novel-miR58 | CGATGTTGGTGAGGTTCAATC | NC_023620.1 | 2319765 | 2319785 | + | 9 | 4 | 0 | 0 |
| Novel-miR59 | CAGTTTAATATCTGATACATG | NC_023625.1 | 4587917 | 4587943 | - | 659 | 26 | 1183 | 1418 |
| Novel-miR60 | ATCTATTGTCTGTAATGTGATTGT | NC_023625.1 | 32452966 | 32452989 | - | 27 | 3 | 0 | 0 |
| Novel-miR61 | AAGGACTAAATGGACCAATCATTT | NC_023616.1 | 1956560 | 1956583 | - | 26 | 6 | 1 | 0 |
| Novel-miR62 | AGGATTTCGTCGATTTGAATCGAG | NC_023624.1 | 37788191 | 37788214 | - | 83 | 6 | 0 | 1 |
| Novel-miR63 | GGGATCTAGAGATAACTACTCACT | NC_023625.1 | 13365220 | 13365241 | + | 13 | 3 | 0 | 0 |
| Novel-miR64 | ATTGATCTTGAACTGTAATTACA | NC_023617.1 | 26243429 | 26243451 | + | 29 | 6 | 2 | 2 |
| Novel-miR65 | TTAAGATCTGCTGGAACCATC | NC_023616.1 | 1767803 | 1767823 | - | 11 | 3 | 0 | 0 |
| Novel-miR66 | AACGTGACTTTGATGTGATAGATA | NC_023621.1 | 39072696 | 39072719 | - | 16 | 2 | 0 | 0 |
| Novel-miR67 | AATGCTTGGACTTGAGCTCCA | NC_023620.1 | 2100133 | 2100153 | - | 25 | 4 | 0 | 0 |

**Supplementary Table 4.** Deseq2 Output for analysis of differentially expressed miRNAs

| **ID miRNA** | **baseMean** | **log2FoldChange** | **lfcSE** | **stat** | **pvalue** | **padj** |
| --- | --- | --- | --- | --- | --- | --- |
| tcc-miR156b | 33.00696242 | 3.176727252 | 0.974528 | 3.259761 | 0.001115 | 0.004258 |
| tcc-miR156e | 5.618752519 | 2.883181402 | 1.421179 | 2.028724 | 0.042486 | NA |
| tcc-miR160a | 42.69854719 | 0.648041602 | 1.035918 | 0.625572 | 0.531596 | 0.637915 |
| tcc-miR160b | 3.25304203 | 1.445054342 | 1.70144 | 0.849313 | 0.395707 | NA |
| tcc-miR162 | 170.2345312 | -1.197973138 | 0.981418 | -1.22066 | 0.222216 | 0.311103 |
| tcc-miR164a | 7.433419669 | -0.931842102 | 1.526039 | -0.61063 | 0.541446 | NA |
| tcc-miR166a | 127.9094547 | -0.091695246 | 0.89642 | -0.10229 | 0.918526 | 0.918526 |
| tcc-miR166c | 2.99304726 | -0.474367151 | 2.055918 | -0.23073 | 0.817523 | NA |
| tcc-miR167a | 17.12729071 | 3.492873417 | 1.15838 | 3.01531 | 0.002567 | 0.008985 |
| tcc-miR171d | 0.829808824 | -0.882047768 | 2.587223 | -0.34092 | 0.73316 | NA |
| tcc-miR172b | 2.189661647 | 0.445191267 | 2.10878 | 0.211113 | 0.832799 | NA |
| tcc-miR172d | 3.395190387 | -2.889706067 | 1.949595 | -1.48221 | 0.138285 | NA |
| tcc-miR390a | 10.91862736 | -0.237425325 | 1.225618 | -0.19372 | 0.846396 | 0.911503 |
| tcc-miR393b | 0.260838538 | 0.745393066 | 4.152137 | 0.17952 | 0.857529 | NA |
| tcc-miR395a | 102.2977102 | 0.113866238 | 0.889673 | 0.127987 | 0.89816 | 0.918526 |
| tcc-miR396a | 55.25705763 | 1.07960002 | 0.918325 | 1.175619 | 0.239747 | 0.324819 |
| tcc-miR396c | 83.83579655 | -2.898413386 | 1.01805 | -2.84703 | 0.004413 | 0.012317 |
| tcc-miR396d | 4.539834245 | 0.431128852 | 1.5899 | 0.271167 | 0.786262 | NA |
| tcc-miR397 | 1.408809669 | -1.609376594 | 2.253022 | -0.71432 | 0.47503 | NA |
| tcc-miR399a | 92.27161933 | -6.174848525 | 1.747326 | -3.53388 | 0.00041 | 0.00215 |
| tcc-miR399g | 0.055893972 | 2.572723425 | 4.91241 | 0.523719 | 0.600474 | NA |
| tcc-miR399i | 7.548266156 | -1.083932555 | 1.503246 | -0.72106 | 0.470872 | NA |
| tcc-miR403a | 110.4033072 | -1.923667136 | 1.029801 | -1.868 | 0.061762 | 0.108084 |
| tcc-miR535 | 48.56812682 | -0.265589108 | 0.962805 | -0.27585 | 0.782664 | 0.86505 |
| tcc-miR827 | 2.980157106 | -0.580826361 | 1.990026 | -0.29187 | 0.770387 | NA |
| Tca-MIR159a | 1.769121036 | 1.149509001 | 2.143472 | 0.536284 | 0.591763 | NA |
| Tca-MIR159b | 126.8180101 | 1.902784467 | 0.910127 | 2.090681 | 0.036557 | 0.08081 |
| Tca-MIR159d | 4817.441914 | -1.762227058 | 0.909927 | -1.93667 | 0.052786 | 0.100773 |
| Tca-MIR165a | 40.80296961 | -2.17535222 | 1.140429 | -1.90749 | 0.056458 | 0.103097 |
| Tca-MIR165g | 6.603599853 | -0.612671382 | 1.538999 | -0.3981 | 0.690558 | NA |
| Tca-MIR170b | 0.378356662 | 0.356112053 | 3.74683 | 0.095044 | 0.92428 | NA |
| Tca-MIR3627a | 12.51849729 | 6.0428505 | 1.350019 | 4.476123 | 7.60E-06 | 7.98E-05 |
| Tca-MIR394a | 1.059114892 | -1.188026626 | 2.409812 | -0.493 | 0.622016 | NA |
| Tca-MIR3954a | 593.8186868 | 2.708907912 | 0.930645 | 2.910787 | 0.003605 | 0.011648 |
| Tca-MIR398a | 26.9691571 | 0.187614517 | 1.198492 | 0.156542 | 0.875606 | 0.918526 |
| Tca-MIR408a | 34.33007544 | -1.470795207 | 1.180296 | -1.24612 | 0.212719 | 0.308076 |
| Tca-MIR472a | 212.2247303 | -2.659700366 | 1.029998 | -2.58224 | 0.009816 | 0.024252 |
| Tca-MIR477a | 2.100184489 | 0.455902014 | 2.058803 | 0.22144 | 0.82475 | NA |
| Tca-MIR477b | 1.791466713 | -1.952773986 | 2.147725 | -0.90923 | 0.363229 | NA |
| Tca-MIR482a | 534.8999233 | -3.865158885 | 0.983013 | -3.93195 | 8.43E-05 | 0.00059 |
| Tca-MIR5225a | 2.799574043 | -0.384717532 | 1.963632 | -0.19592 | 0.844672 | NA |
| Tca-MIR530a | 0.943026565 | -0.986790217 | 2.52096 | -0.39143 | 0.695476 | NA |
| Tca-MIRN1 | 0.200644184 | 1.222687237 | 4.207564 | 0.290593 | 0.771363 | NA |
| Tca-MIRN10 | 8.305565509 | -0.377679799 | 1.38423 | -0.27284 | 0.784973 | NA |
| Tca-MIRN2 | 0.480114046 | -0.10184413 | 3.069044 | -0.03318 | 0.973528 | NA |
| Tca-MIRN3 | 2.39626985 | -2.402942177 | 2.061212 | -1.16579 | 0.243699 | NA |
| Tca-MIRN5 | 29.54397651 | -1.892588362 | 1.204573 | -1.57117 | 0.116143 | 0.180667 |
| Tca-MIRN7 | 9.150549042 | -1.459482365 | 1.479853 | -0.98623 | 0.324018 | 0.412386 |
| Tca-MIRN7068a | 191.4055245 | -2.358064787 | 1.072685 | -2.19828 | 0.027929 | 0.065168 |
| Tca-MIRN9 | 253.5181093 | -3.173302709 | 1.122328 | -2.82743 | 0.004692 | 0.012317 |
| miRNA_novel_1 | 1137.454905 | -1.736590067 | 0.871678 | -1.99224 | 0.046345 | 0.097325 |
| miRNA_novel_2 | 85.88918197 | -4.492336637 | 1.344816 | -3.34048 | 0.000836 | 0.003903 |
| miRNA_novel_3 | 12.12210538 | -1.222456374 | 1.33807 | -0.9136 | 0.360929 | 0.445853 |
| miRNA_novel_4 | 62.10400923 | -0.421335685 | 0.944704 | -0.446 | 0.655599 | 0.744193 |
| miRNA_novel_5 | 95.32704354 | -1.593746606 | 0.913585 | -1.7445 | 0.081072 | 0.133514 |
| miRNA_novel_6 | 819.076669 | 4.483705239 | 0.890853 | 5.033049 | 4.83E-07 | 6.76E-06 |
| miRNA_novel_7 | 6.244331204 | -3.748913645 | 1.857898 | -2.01783 | 0.043609 | NA |
| miRNA_novel_8 | 16.4028216 | -1.747320284 | 1.314927 | -1.32883 | 0.183903 | 0.275854 |
| miRNA_novel_9 | 15.72476065 | -5.08691538 | 1.788303 | -2.84455 | 0.004447 | 0.012317 |
| miRNA_novel_10 | 4.511640041 | -3.316861538 | 1.934205 | -1.71484 | 0.086374 | NA |
| miRNA_novel_11 | 4.459777719 | 0.182193756 | 1.639609 | 0.11112 | 0.911521 | NA |
| miRNA_novel_12 | 4.530260373 | -3.292062326 | 1.891834 | -1.74014 | 0.081834 | NA |
| miRNA_novel_13 | 3.899443088 | -1.080472507 | 1.984783 | -0.54438 | 0.586181 | NA |
| miRNA_novel_14 | 5.140793688 | -3.478910778 | 1.867594 | -1.86278 | 0.062494 | NA |
| miRNA_novel_15 | 2.573982328 | -2.497200913 | 2.027516 | -1.23166 | 0.218078 | NA |
| miRNA_novel_16 | 7.445067983 | -1.0283104 | 1.535071 | -0.66988 | 0.502935 | NA |
| miRNA_novel_17 | 3.574074923 | 0.948563079 | 1.710278 | 0.554625 | 0.579151 | NA |
| miRNA_novel_18 | 106.3987551 | 5.527143609 | 0.903083 | 6.120304 | 9.34E-10 | 1.96E-08 |
| miRNA_novel_19 | 3.192261647 | -0.66853873 | 1.935521 | -0.34541 | 0.72979 | NA |
| miRNA_novel_20 | 3.87673423 | -3.06984444 | 1.919735 | -1.5991 | 0.109799 | NA |
| miRNA_novel_21 | 0.955927711 | -1.071782887 | 2.477366 | -0.43263 | 0.665284 | NA |
| miRNA_novel_22 | 0.788245793 | -0.780456749 | 2.605446 | -0.29955 | 0.764522 | NA |
| miRNA_novel_23 | 18.29166646 | -1.430357498 | 1.258076 | -1.13694 | 0.255563 | 0.335427 |
| miRNA_novel_24 | 0.434250634 | 0.152219063 | 3.203849 | 0.047511 | 0.962106 | NA |
| miRNA_novel_25 | 0.77964503 | -0.709096983 | 2.653859 | -0.26719 | 0.789319 | NA |
| miRNA_novel_26 | 2.485712118 | -0.142994472 | 2.092358 | -0.06834 | 0.945514 | NA |
| miRNA_novel_27 | 2.92080652 | -2.65467385 | 1.990228 | -1.33385 | 0.182252 | NA |
| miRNA_novel_28 | 1.537565302 | 2.58784683 | 2.274266 | 1.137882 | 0.25517 | NA |
| miRNA_novel_29 | 6.940920724 | 2.534055553 | 1.396181 | 1.814991 | 0.069525 | NA |
| miRNA_novel_30 | 6.442245135 | 0.49207035 | 1.401491 | 0.351105 | 0.72551 | NA |
| miRNA_novel_31 | 1.265489254 | -1.403952224 | 2.40317 | -0.58421 | 0.55908 | NA |
| miRNA_novel_32 | 4.804011049 | -3.411091514 | 1.967876 | -1.73339 | 0.083027 | NA |
| miRNA_novel_33 | 2.489426471 | -2.458887858 | 2.051883 | -1.19836 | 0.230778 | NA |
| miRNA_novel_34 | 31.81984198 | 4.397920109 | 1.06156 | 4.142882 | 3.43E-05 | 0.000288 |
| miRNA_novel_35 | 0.970258653 | -1.0736366 | 2.461532 | -0.43617 | 0.662716 | NA |
| miRNA_novel_36 | 2.490856267 | -2.43626864 | 2.030689 | -1.19972 | 0.230246 | NA |
| miRNA_novel_37 | 3.131024576 | 2.378744508 | 1.738494 | 1.368279 | 0.171225 | NA |
| miRNA_novel_38 | 1.026152626 | -1.158984785 | 2.426752 | -0.47759 | 0.632944 | NA |
| miRNA_novel_39 | 24.03148262 | -5.710880189 | 1.747414 | -3.26819 | 0.001082 | 0.004258 |
| miRNA_novel_40 | 0.988889977 | -1.102665533 | 2.449468 | -0.45017 | 0.652591 | NA |
| miRNA_novel_41 | 1.516883261 | 1.84189547 | 2.23787 | 0.823058 | 0.410475 | NA |
| miRNA_novel_42 | 372.9288666 | 3.662431924 | 0.955904 | 3.831381 | 0.000127 | 0.000765 |
| miRNA_novel_43 | 1.847946714 | 0.864264796 | 2.120299 | 0.407615 | 0.683557 | NA |
| miRNA_novel_44 | 16.33436768 | -0.675832834 | 1.150198 | -0.58758 | 0.556815 | 0.649617 |
| miRNA_novel_45 | 4.922958969 | -3.436032798 | 1.895408 | -1.81282 | 0.06986 | NA |
| miRNA_novel_46 | 0.620563876 | -0.412704666 | 2.799513 | -0.14742 | 0.8828 | NA |
| miRNA_novel_47 | 17.59919653 | -3.701823586 | 1.911254 | -1.93686 | 0.052763 | 0.100773 |
| miRNA_novel_48 | 11.77122756 | -3.077693446 | 1.773373 | -1.7355 | 0.082652 | 0.133514 |
| miRNA_novel_49 | 2.205656226 | -2.27416048 | 2.075542 | -1.09569 | 0.273212 | NA |
| miRNA_novel_50 | 1.067715656 | -1.237059937 | 2.413656 | -0.51253 | 0.608283 | NA |
| miRNA_novel_51 | 8.269310859 | 0.673675802 | 1.292921 | 0.52105 | 0.602332 | NA |
| miRNA_novel_52 | 3.955559909 | -3.103265986 | 1.914437 | -1.62098 | 0.105022 | NA |
| miRNA_novel_53 | 0.802576736 | -0.781348725 | 2.596195 | -0.30096 | 0.763446 | NA |
| miRNA_novel_54 | 1.381577581 | -1.552512328 | 2.285609 | -0.67926 | 0.496976 | NA |
| miRNA_novel_55 | 6.437944753 | 0.501892571 | 1.389829 | 0.361118 | 0.718011 | NA |
| miRNA_novel_56 | 1.516297232 | -1.70895534 | 2.220346 | -0.76968 | 0.44149 | NA |
| miRNA_novel_57 | 3.584105484 | 0.98725984 | 1.665435 | 0.592794 | 0.553319 | NA |
| miRNA_novel_58 | 0.672157466 | -0.503340867 | 2.751885 | -0.18291 | 0.85487 | NA |
| miRNA_novel_59 | 3256.586447 | 7.689200881 | 0.930303 | 8.265262 | 1.39E-16 | 5.85E-15 |
| miRNA_novel_60 | 0.881402414 | -0.949688683 | 2.528503 | -0.37559 | 0.707219 | NA |
| miRNA_novel_61 | 2.559417545 | 0.052115242 | 1.994701 | 0.026127 | 0.979156 | NA |
| miRNA_novel_62 | 3.488933037 | -0.917992645 | 1.923065 | -0.47736 | 0.633106 | NA |
| miRNA_novel_63 | 0.620563876 | -0.412704666 | 2.799513 | -0.14742 | 0.8828 | NA |
| miRNA_novel_64 | 6.305240928 | 1.9783716 | 1.328319 | 1.48938 | 0.136387 | NA |
| miRNA_novel_65 | 0.583301228 | -0.316134286 | 2.85742 | -0.11064 | 0.911905 | NA |
| miRNA_novel_66 | 0.550338961 | -0.266096468 | 2.907281 | -0.09153 | 0.927073 | NA |
| miRNA_novel_67 | 0.970258653 | -1.0736366 | 2.461532 | -0.43617 | 0.662716 | NA |


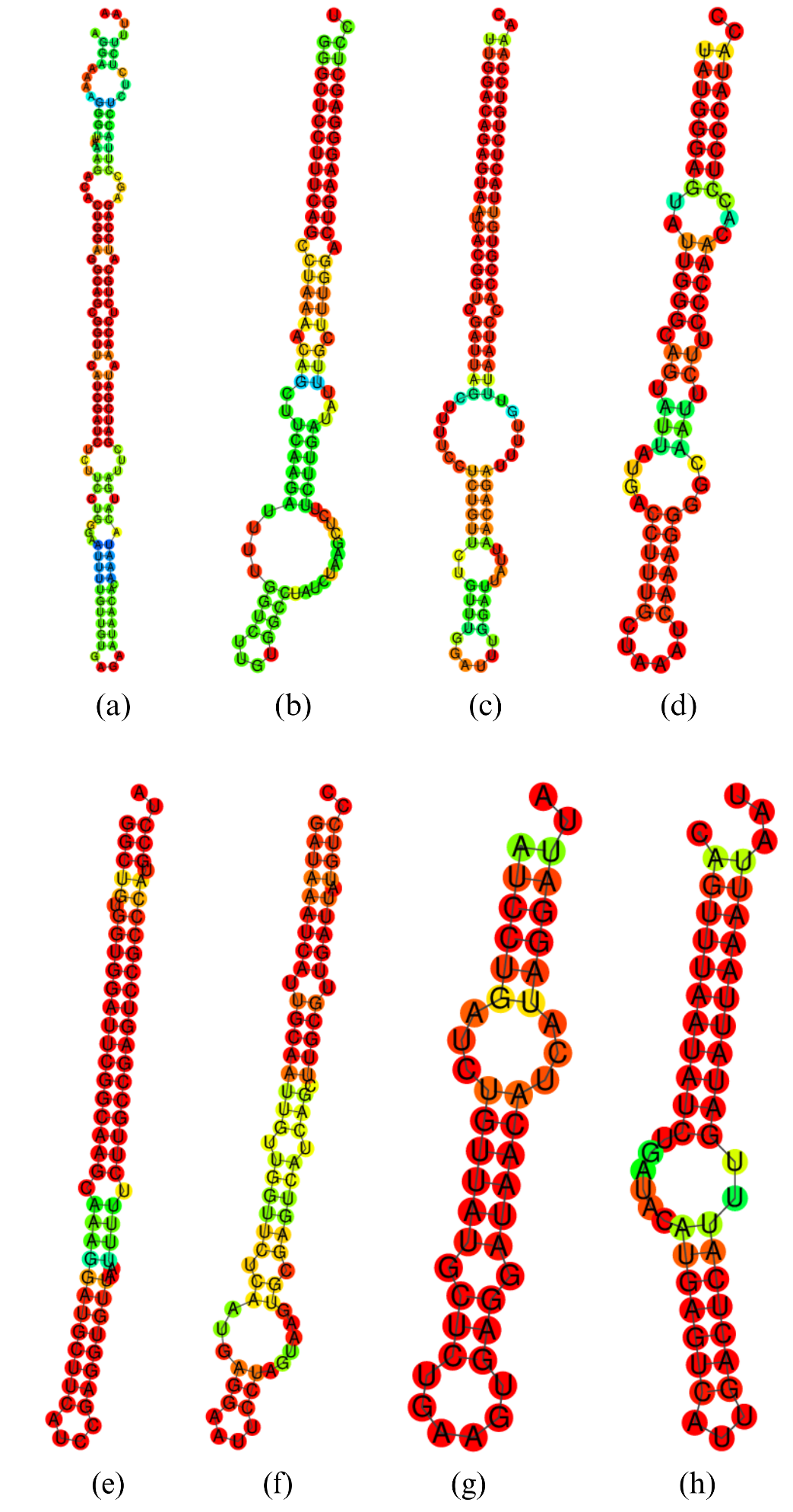


**Supplementary Figure 1. S**econdary structure of known and novel pre-miRNAs with the highest abundance. (a) tcc-miR162; (b) tca-MIR159d; (c) Tca-MIR3954a; (d) Tca-MIR482a; (e) Novel-miR1; (f) Novel-miR6; (g) Novel-miR42; and (h) Novel-miR59.
